# Fabrication and performance evaluation of new nanocomposite membranes based on sulfonated poly(phthalazinone ether ketone) for PEM fuel cells

**DOI:** 10.1039/c9ra08893h

**Published:** 2020-01-15

**Authors:** Khadijeh Hooshyari, Samira Heydari, Mehran Javanbakht, Hossein Beydaghi, Morteza Enhessari

**Affiliations:** Department of Applied Chemistry, Faculty of Chemistry, Urmia University Urmia Iran Kh.Hooshyari@urmia.ac.ir; Department of Chemistry, Amirkabir University of Technology Tehran Iran; Solar Cell and Fuel Cell Lab, Renewable Energy Research Center, Amirkabir University of Technology Tehran Iran; Graphene Labs, Istituto Italiano di Tecnologia 16163 Genova Italy; Department of Chemistry, Naragh Branch, Islamic Azad University Naragh Iran

## Abstract

The purpose of this work is to enhance the proton conductivity and fuel cell performance of sulfonated poly(phthalazinone ether ketone) (SPPEK) as a proton exchange membrane through the application of SrTiO_3_ perovskite nanoparticles. Nanocomposite membranes based on SPPEK and SrTiO_3_ perovskite nanoparticles were prepared *via* a casting method. The highest proton conductivity of nanocomposite membranes obtained was 120 mS cm^−1^ at 90 °C and 95% RH. These enhancements could be related to the hygroscopic structure of SrTiO_3_ perovskite nanoparticles and the formation of hydrogen bonds between nanoparticles and water molecules. The satisfactory power density, 0.41 W cm^−2^ at 0.5 V and 85 °C, of the nanocomposite membrane (5 wt% content of nanoparticles) confirms their potential for application in the PEM fuel cells.

## Abstract 2

The purpose of this work is to enhance the proton conductivity and fuel cell performance of sulfonated poly(phthalazinone ether ketone) (SPPEK) as a proton exchange membrane through the application of SrTiO_3_ perovskite nanoparticles.

## Graphical abstract

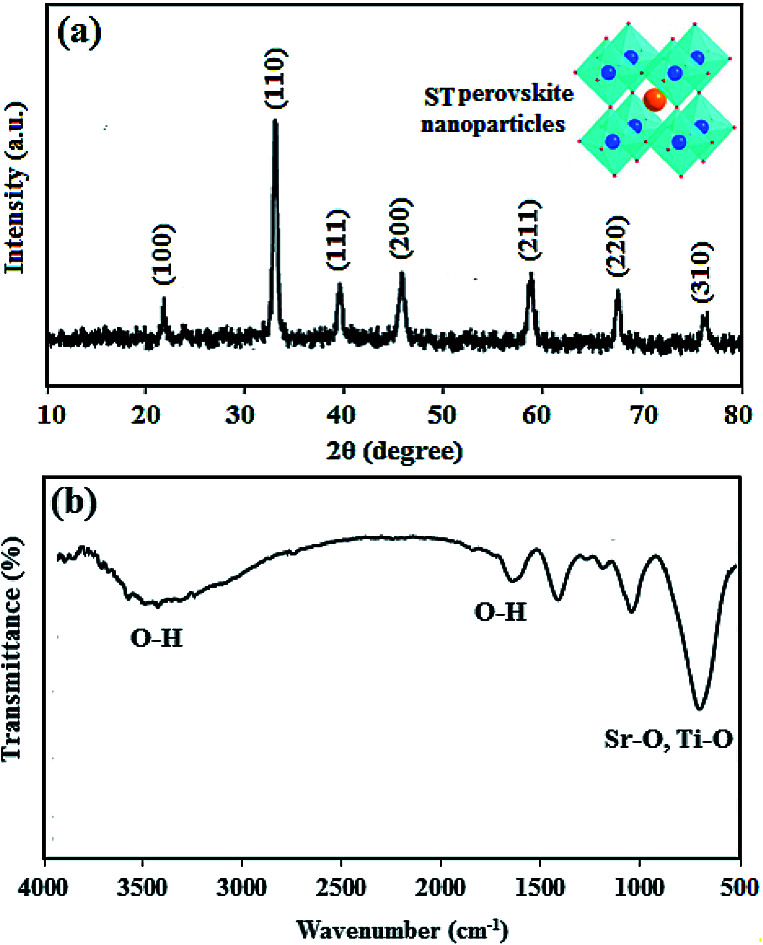

## 1. Introduction

Burning fossil fuels in order to produce energy is a threat to the environment, especially the ozone layer. Today the world is looking for renewable sources to supply energy.^1,2^ The fuel cell is one of the best alternatives as a clean source for this purpose. Hydrogen is an environment friendly fuel because the product of its combustion is pure water. Fuel cells transform chemical energy to electrical energy in one step. In most cases, a catalytic material accelerates this phenomenon.^3–5^ Among the different types of fuel cells, the proton exchange membrane (PEM) fuel cells are the most applicable, because of their low weight and volume, low operating temperature and ease of fuel storage.^6–9^ The structure and performance of PEMs are the most important issues of fuel cell science, since they have significant effects on ohmic drop, electrode polarization and total performance of PEM fuel cells. A qualified membrane should have sufficient proton conductivity, good water uptake, suitable thermal stability, high mechanical strength and chemical resistance. Moreover, it should prevent the crossover of reactant gases.^10–12^ Up to now, Nafion has been the most widely used membrane. It is a kind of perfluorosulfonate ionomer membranes, a polymer with the noted properties as a qualified membrane, which has sulfonic acid groups in its side chain. However, Nafion is so expensive and has low proton conductivity in low relative humidity.^13^ On the other hand, because of proton transport mechanism, the proton conductivity is vigorously affected by the presence of water, which means that the membrane must be hydrated to obtain maximum ability of transporting protons. Therefore, Nafion has suitable proton conductivity at temperatures under the water boiling point but at higher temperatures, a significant decline would be observed in conductivity and consequently in PEM fuel cells performance.^14,15^ Many efforts had been made in order to improve these defects, but these emprises would not result in cheaper membranes.

Many efforts have been made to find a substitute for Nafion membrane. Sulfonated non-fluorinated polymers such as: sulphonated poly(ether ether ketone),^16,17^ sulphonated poly(ether ketone), sulphonated poly(sulfone),^18^ sulfonated poly(vinylidene fluoride-*co*-hexafluoro propylene),^19^ sulphonated polybenzimidazole, sulphonated polyimide have been studied.^20,21^ These polymers have suitable mechanical, chemical, thermal and oxidative stability.^22,23^ Sulfonated poly(phthalazinone ether ketone) (SPPEK) is one of the sulphonated polymers with eligible properties for use as PEMs. It is a thermoplastic polymer with high glass transition temperature that has excellent thermal and mechanical stability.^24,25^ Nitrogen atoms in the SPPEK backbone and high content of sulfonic acid groups lead to high proton conductivity of this polymer. The presence of sulfonic acid functional groups on the side chains of aromatic polymers cause the ionic clusters to be gathered. Consequently, the hydrophilic and hydrophobic sites separate from each other and this phenomenon leads to better proton conductivity.^26^ Furthermore, SPPEK is not expensive in comparison with Nafion.^27–29^ Sulfonic acid groups can efficiently cause improvement in the proton conductivity of sulphonated polymers *via* Grotthuss mechanism. But on the contrary, dimensional instability often occurs in membranes with high content of sulfonic acid groups while swelling. Because the membrane will experience hot press in the process of producing membrane electrode assembly, then it should work at a humidified hot condition in the fuel cell, it is so important to save the mechanical stability, simultaneous with having appropriate proton conductivity. Therefore, the membrane must have a suitable dimensional stability. Inorganic materials can improve mechanical properties of the membrane.^30,31^ Also, doping with hygroscopic materials can improve membrane's water uptake ability and consequently its proton conductivity. SiO_2_, ZrO_2_ and TiO_2_ are hygroscopic materials which adsorb water and cause better water uptake and proton exchange ability.^32–34^ Proton transfer occurs easier if more water molecules would be captured in the polymer structure. Many organic–inorganic membranes have been manufactured.^35^ Du *et al.* fabricated a composite membrane using SPEEK doped with SiO_2_ sulfuric acid, they were successful to increase the SPEEK proton conductivity with the value of 18.6%.^36^ Yu-Huei Su *et al.* mixed SPPEK and SiO_2_ to form the PEM composites. Thermal and mechanical properties and dimensional stability of these membranes have been improved.^37^ Recently, different nanocomposite membranes based on barium zirconate nanoparticles,^38–40^ strontium cerate nanoparticles,^41^ lanthanum cerate^42^ and iron titanate nanoparticles^43–45^ have been investigated.

A very stable type of oxide with the schematic formula of ABO_3_, which is named perovskite, can be a suitable choice for this purpose. In the ABO_3_ formula of perovskites, A (A cations are 12-coordinated by oxygen and have a large ionic radius with similar size as the oxygen ion) and B (B cations are 6-coordinated by oxygen and have a small ionic radius, which situated in the octahedral holes between the closed pack AO-layers) are cations which are appropriate in perovskite structures. Perovskite groups show high stability due to the special orientation of atoms. Research on perovskite-type (ABO_3_) pure materials has been enhanced in recent years due to their smart high proton conductivity under wet atmospheres at high temperatures (1000 °C).^46,47^ The perovskite nanoparticles have high thermal, mechanical, and chemical stabilities. The perovskites can be insulators, semiconductors, superconductors, and ionic conductors.^48^ Different types of large and small ions can be incorporated in perovskite structure. So these types of oxides have the ability to contain diverse alignment of chemicals. Consequently, the hallow spaces are formed easily in the crystalline structure of this component. Perovskites have sufficient properties for proton conduction because of free spaces for oxygen sites capture water molecules, facilitating proton mobility.

The most important perovskite from the titanate family is strontium titanate SrTiO_3_ (ST). The ST nanoparticle with perovskite-type cubic structure is a promising material for using in solid oxide fuel cells, capacitors, photo-catalysis, magneto-hydrodynamic power generation and oxygen sensors.^49^ The perovskite materials such as BaCeO_3_, BaZrO_3_, SrCeO_3_ and SrTiO_3_ have good proton conductivity (0.01–10 mS cm^−1^) in temperature range of 200–1200 °C.^50^ Behaviors of defects in strontium titanate (SrTiO_3−*δ*_), one of the most common perovskite-type metal oxides, have been studied during last 40 years. The oxygen vacancy would be formed in oxygen-deficient ABO_3−*δ*_ as a nonstoichiometric perovskite, where *δ* expresses the number of deficient atoms per unit formula (oxygen vacancy spaces). The oxygen vacancy spaces in the perovskite nanoparticles raise the proton conductivity because of its role as hydrogen traps.

In this work, we produced nanocomposite membranes based on SPPEK doped with SrTiO_3_ (ST) perovskite nanoparticles. The ratio of nanoparticles had been changed and the trend of properties changes was investigated at different temperatures for the first time. The results showed that mechanical stability, water uptake, proton conductivity and single fuel cell test performance of the nanocomposite membranes improved. The SrTiO_3_ perovskite nanoparticles caused an enhancement in water uptake and proton conductivity of membranes by forming hydrogen bonds and trapping water molecules in the membrane matrix. Mechanical stability of the nanocomposite membranes enhanced because of the increase of inorganic content and also the strong hydrogen bonds between SO_3_H groups of the SPPEK and ST perovskite nanoparticles. As expected, by improving water uptake and proton conductivity of the membranes, they showed better fuel cell performance and supplied more power density in the presence of nanoparticles.

## 2. Experimental

### 2.1. Materials

Poly(phthalazinone ether ketone), PPEK, (*M*_w_ = 28 800 g mol^−1^, *M*_n_ = 10 300) was supplied by Fumatech Company. Dimethylacetamide (DMAc), 2-propanol and glycerol were supplied from Merck Company. Carbon Vulcan and Pt/C 20% powder were obtained from Electrochem Company. Nafion 117 membrane, Nafion 5% solution and Teflon solution were supplied by Dupont. SrTiO_3_ perovskite nanoparticle with the size of 50 nm was used as inorganic additive.

### 2.2. Synthesis of SrTiO_3_ perovskite nanoparticles

The SrTiO_3_ (ST) perovskite nanoparticles were simply synthesized according to previously described method.^51^ 0.5982 g SrO_2_, 0.3994 g TiO_2_ and 1.5 g of NaCl and KCl (a mixture with equal molar ratios) were blended thoroughly in a carnelian mortar. Afterward, the homogenous mixture was heated from ambient temperature to 700 °C for 12 hours in a corundum pounder and was leaved to become cool. After reaching to room temperature, the probable contaminants were washed by 1 M solution of HNO_3_ in water. The solid residue was dried at 80 °C and finally, ST perovskite nanoparticles were gained in the shape of a white powder.

### 2.3. Sulfonation of PPEK

The SPPEK was prepared by direct sulfonation of PPEK with sulfuric acid according to the following procedure; first, 2 g of PPEK powder was added into 20 mL concentrated sulfuric acid and was stirred for 1 hour at ambient temperature. The prepared solution was stirred for 4 hours under the temperature of 60 °C. Then, the solution was poured into ice water simultaneous with shaking. The residue powder was washed by deionized water for several times to be neutralized. Finally, the SPPEK powder was dried in a vacuum oven at 70 °C for 24 hours.

The introduction of sulfonic acid groups on PPEK backbone causes the appearance of a significant signal at 8.30 ppm, which is related to the aromatic proton (H_E_) next to SO_3_H. DS was calculated by the following eqn (1):1
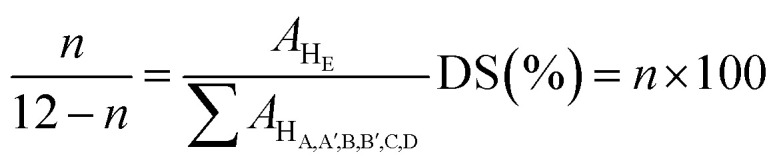
where, *n* is the number of H_E_ per repeat unit. The DS of the SPPEK was determined by eqn (1) and titration method. The results indicate that both techniques gave identical results. The DS of SPPEK powder was obtained 68%.

### 2.4. Preparation of nanocomposite membranes

Nanocomposite membranes were fabricated *via* solution casting method. Appropriate amount of SPPEK was dissolved in DMAc at 60 °C and 6 h stirring to obtain a homogeneous solution (15 wt%). The certain amounts of the ST perovskite nanoparticles (1, 3, 5, 7 and 9 wt%) were dispersed in DMAc using an ultrasonic probe and then were added to the above SPPEK solution while stirring in order to obtain a homogenous solution. The prepared quite viscose solution was cast onto a glass plate with applicator and dried at 25 °C for 12 h, 70 °C for 12 h and 120 °C for 2 h to remove any residual DMAc. Finally, the glass plate was soaked in water in order to separate the obtained membrane. The thicknesses of prepared membranes were ranged between 80 and 100 μm. The prepared nanocomposite membranes based on SPPEK and ST perovskite nanoparticles were named SPP-ST_*x*_. The value of *x* in SPP-ST_*x*_ nanocomposite membranes was assigned for the *x* wt% of the ST perovskite nanoparticles *vs.* weight of dry polymer.

### 2.5. Apparatus

Biologic fuel cell tester, series of FCT-150s, was used to obtain polarization curves. Autolab potentiostat-galvanostat PGstat 302N was used to perform electrochemical tests. ATR-FTIR spectra of the membranes were performed with a Bruker Equinox 55 (600–4000 cm^−1^ and resolution of 4 cm^−1^) at the room temperature. Field emission scanning electron microscopy (FE-SEM), Carl Zeiss electronic microscope, was used to analyze membrane's morphology and energy dispersive X-ray spectroscopy (EDX) was used to confirm the homogeneous distribution of the nanoparticles in the nanocomposite membranes matrix. Tensile strength test was performed with the purpose of exploring mechanical stability of the membrane using Instron-5566 instrument. Membranes were cut to strips with dimensions of 5 and 50 mm, and stretched with the speed of 2 mm min^−1^. Thermal stability was investigated at ambient pressure from 30 to 700 °C with the rate of 20 °C min^−1^. H-NMR device model Oxford 600 MHz was used to investigate the structure of the SPPEK.

### 2.6. Characterization of membranes

#### 2.6.1. Water uptake and membrane swelling

The water uptake and membrane swelling have a enormous influence on proton conductivity and mechanical stability of prepared membranes. Cut pieces from each of the membranes were immersed in deionized water for 24 h. After taken out, the surface adsorbed water was cleaned and the wet weight was measured at different temperatures. Then the membrane was heated at 80 °C for 24 h to obtain a dry weigh. Finally, the water uptake (WU) was calculated *via*eqn (2):2
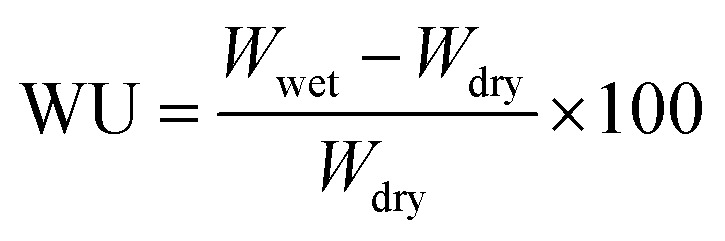
*W*_wet_ and *W*_dry_ represent the weight of hydrated membrane and the weight of dry membrane, respectively.

In a process similar to that applied for detecting the water uptake, the thicknesses of the dry and wet membranes were measured with using thickness gauge. The swelling ratios (SW) of the membranes were obtained from eqn (3):3
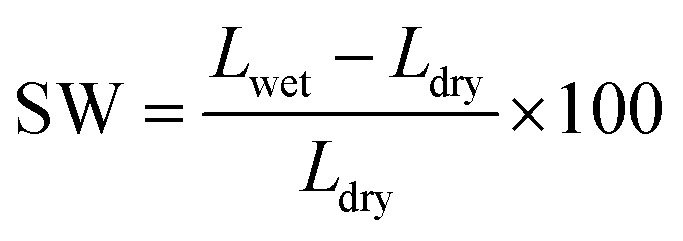
where, *L*_wet_ represents the thicknesses of wet membranes obtained after immersing in deionized water for 24 h, and *L*_dry_ is the thickness of the dry membranes.

#### 2.6.2. Proton conductivity and ion exchange capacity (IEC)

Proton conductivity of membranes was measured using electrochemical impedance spectroscopy (EIS) technique. The proton conductivity measurement of the membranes was taken at the frequency range of 100 Hz to MHz and signal amplitude of 50 mV by a PGSTAT303N Autolab. Proton conductivity was measured according to eqn (4):4
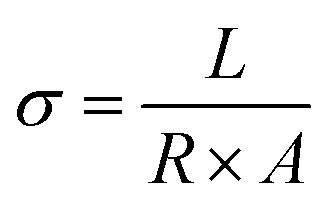
where *σ* is the proton conductivity (S cm^−1^), *L* is the membrane thickness (cm), *A* is the cross-sectional area of the membrane (cm^2^) and *R* is resistance of the membrane (Ω), which obtain from the Nyquist plot at the highest frequency.

Activation energy (*E*_a_) was obtained using Arrhenius eqn (5):5
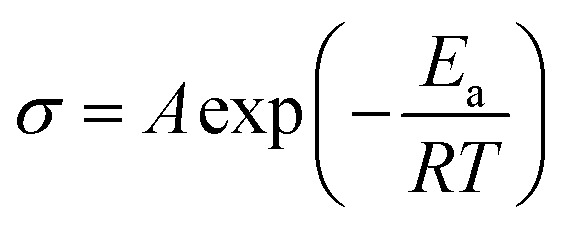
where, *A* is the pre-exponential factor, *R* is gas constant (8.314 J mol^−1^ K^−1^); *T* is temperature (K). The activation energy values were obtained from the slope of the Arrhenius plots.

For determining the IEC, the membranes were weighed, then, immersed in a saturated NaCl solution for thorough substitution of H^+^ by Na^+^ ions of NaCl. After that, the released H^+^ ions from the polymer to the salty solution were titrated by NaOH in the presence of phenolphthalein as indicator. The eqn (6) was used to calculate the IEC of the membranes:6
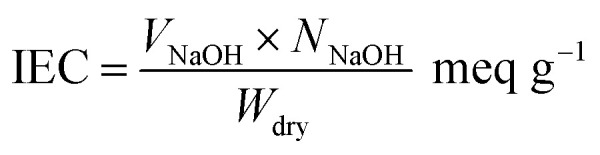
*V*_NaOH_ and *N*_NaOH_ are the volume and normality of NaOH and *W*_dry_ is the weight of dry membrane.

#### 2.6.3. Oxidative stability

In order to investigate chemical stability of the membranes, the membranes immersed in Fenton's reagent (3% H_2_O_2_ solution and 4 ppm Fe^2+^) at 70 °C for 4 h. Fe^2+^ was added as FeSO_4_·7H_2_O for accelerating generation of hydroxide radicals. The prepared membranes were placed in Fenton reagent until the membrane began to break.

#### 2.6.4. Single fuel cell test

The prepared membranes were used for fabrication the MEA. To prepare the catalyst layer, a 20 wt% Pt–C powder (Pt loading of 0.5 mg cm^−2^) was used. The process of MEA production is as follow. First, a certain amount of Pt–C powder was dissolved in mixture of water/isopropanol alcohol (1 : 9) to prepare the catalyst ink. Then a 5 wt% of Nafion solution was added into the above solution. The weight ratio of Nafion to platinum in the Pt/C powder in the ink is 1 : 1. The catalyst ink was painted on the carbon cloth containing microporous layer at 120 °C and 50 kg cm^−2^ pressure for 5 min to prepare the MEAs. For activation processes, the temperature of anode, cathode and humidifiers (RH range: 60–95%) was increased step by step until the temperature reaches 80 °C for all issues. The cell was put in 0.6 V at a constant current for 6 h until the cell was activated fully.

## 3. Results and discussion

### 3.1. ST perovskite nanoparticles characterization

**Fig. 1:**
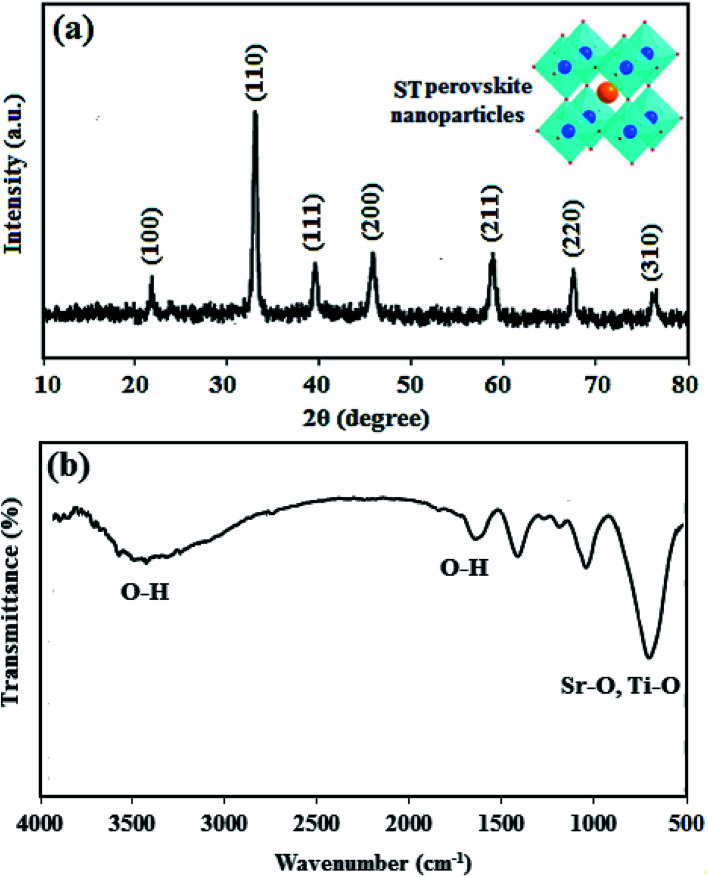
XRD pattern (a) and FTIR spectrum (b) of ST perovskite nanoparticles.

XRD pattern and FTIR spectra of ST perovskite nanoparticles were shown in Fig. 1. The XRD pattern (Fig. 1(a)) indicates the crystal of the as-synthesized ST perovskite nanoparticles appears a typical perovskite structure of the cubic symmetry. The diffraction peaks of the ST perovskite nanoparticles were located at 22.7°, 32.3°, 39.9°, 46.3°, 57.6°, 67.5°, and 77.1°, corresponding to the (100), (110), (111), (200), (211), (220), and (310) crystal planes, respectively, of cubic perovskite ST nanoparticles consist of single phase (according to JCPDS No.74-1296).^49^ However, the perfect perovskite nanoparticles structure is scarcely found at ambient temperature/pressure due to the strict constraints placed on the ionic sizes of A, B and O in ABO_3_ structure of perovskites.^52^ The Goldschmidt tolerance factor, *t*, is determined from ionic radii, *r*_A_, *r*_B_ and *r*_O_ as following: *t* = (*r*_A_ + *r*_O_)/√2(*r*_B_ + *r*_O_). The Goldschmidt tolerance factor is based on the geometrical packing of charge spheres and for the ideally packed perovskite structure (simple cubic) represented with *t* = 1. However, a large number of perovskite structures are distorted to orthorhombic, rhombohedral or tetragonal which can be approximated as cubic with *t* deviated from 1. In most cases, *t* varies between 0.75 and 1.^53,54^ These approximated cubic structures due to tensile strain and lower activation energies increase proton transfer. The average crystallite size of ST perovskite nanoparticles was determined by Deby–Scherer's eqn (6) (in which is the X-ray wavelength in nanometer, *θ* is ½ the diffraction angle and *β* is the full width half maximum in radian eqn (7)) and obtained about 50 nm. The ST perovskite nanoparticles with particle size of 50 nm have the high specific surface area that makes strong hydrogen bonding with water in the nanocomposite membrane.7
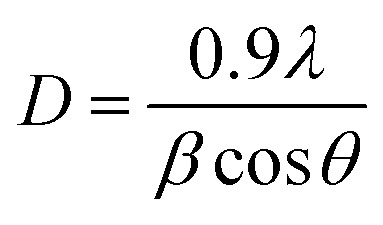


From Fig. 1(b), at low frequencies, the typical tensile vibration of metal–oxygen at 763–550 cm^−1^ is the vibrational region of Sr–O, Ti–O in titanate compounds, which confirms the presence of ST perovskite nanoparticles. The bands at 3435 cm^−1^ and 1630 cm^−1^ was assigned to the O–H vibration, which confirm the existence of O–H groups according to the adsorbed to the water on the ST perovskite nanoparticles. The O–H groups in ST perovskite nanoparticles confirm the water uptake adsorption ability, which can increase the proton conductivity of nanocomposite membrane.

### 3.2. SPP-ST_*x*_ nanocomposite membranes characterization

#### 3.2.1. ATR analysis

**Fig. 2:**
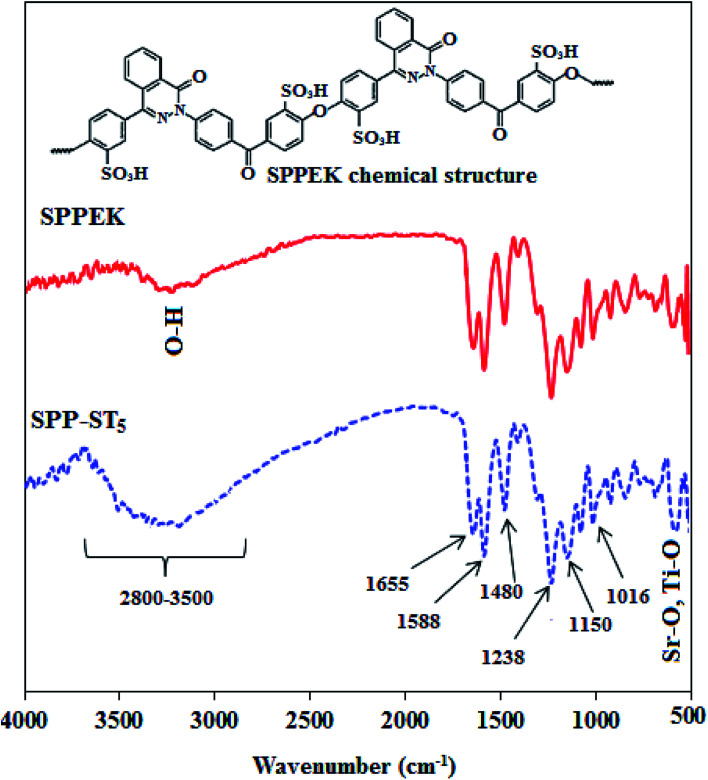
ATR spectra of SPPEK and SPP-ST_5_ membranes.

The ATR spectra of SPP-ST_5_ nanocomposite membranes are shown in Fig. 2.

The broad adsorption near 3400 cm^−1^ is attributed to O–H band in SO_3_H groups. The carbonyl band is seen in 1655 cm^−1^and the C=N band is seen in 1588 cm^−1^ in the spectrum. Two peaks in 1480 and 1238 cm^−1^ refer to the conjugated carbons in SPPEK, C–C and C–O–C bands, respectively.^55^ The peaks in approximately 1020 and 1100 cm^−1^ illustrate the symmetric and asymmetric stretching vibration of S=O=S in SO_3_H groups, confirmed the addition of sulfonated groups in the structure of polymer.^55,56^ As shown in Fig. 2, after adding ST perovskite nanoparticles to SPPEK, the intensity of O–H group related peaks was increased which is related to hydrophilic nature of ST perovskite nanoparticles in the SPP-ST_5_ nanocomposite membranes structure. In addition, the peak that appeared around 763–550 cm^−1^ in SPP-ST_5_ nanocomposite membrane is may be assigned to presence of Sr–O and Ti–O vibration in the structure of nanocomposite membrane.

#### 3.2.2. Proton conductivity

**Fig. 3:**
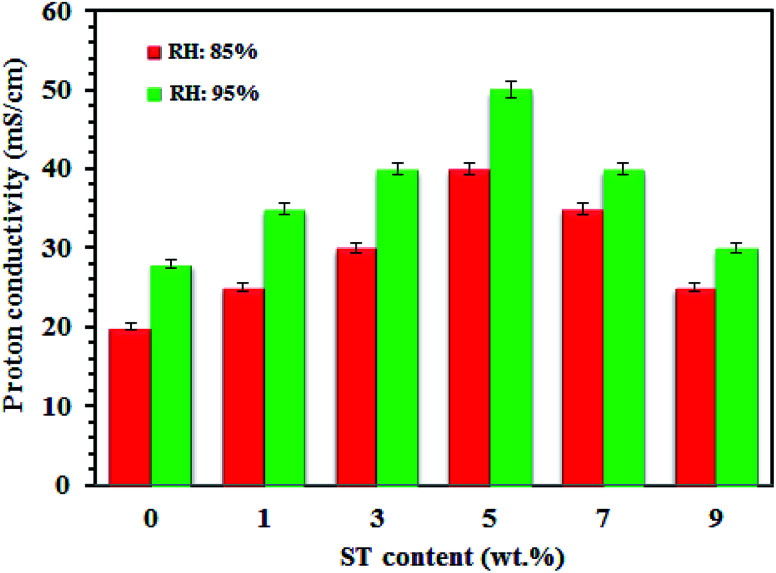
Proton conductivity plots of SPP-ST_*x*_ nanocomposite membranes at 25 °C and different RH.

Fig. 3 shows proton conductivity of SPP-ST_*x*_ nanocomposite membranes at 25 °C and different RH. The SPP-ST_*x*_ nanocomposite membranes showed high proton conductivity compared with pure SPPEK membrane. It is due to the existence of the high hydrophilic ST perovskite nanoparticles *via* oxygen vacancies in the nanocomposite membranes. Proton conduction in SPP-ST_*x*_ nanocomposite membrane is affected by the chemical properties of SPPEK membrane matrix and the ST perovskite nanoparticles. The quality of proton conductivity relates to water presence, ST perovskite nanoparticles and the SO_3_H groups in the SPPEK membrane matrix. The ordered arrangement of oxygen vacancies of the ST perovskite nanoparticles increases hydrogen bonding interactions in the SPP-ST_*x*_ nanocomposite membranes compared to the pure SPPEK membrane. The SPP-ST_5_ nanocomposite membrane showed the highest proton conductivity (50 mS cm^−1^ at 25 °C and 95% RH) compared with other SPP-ST_*x*_ nanocomposite membranes.

Water molecules in the nanocomposite membranes cause the transportation of protons in two mechanisms by forming a network of hydrogen bonds: hopping (Grotthuss) mechanism is performed by bonded H_2_O in which the protons jump from one molecule to another molecule. The other one, the vehicle mechanism occurs by free H_2_O molecules, and proton transport refers to different forms of H^+^ such as; H_3_O^+^ and H_5_O_2_^+^. The SO_3_H groups in the SPPEK membrane and oxygen vacancies of the ST perovskite nanoparticles capture water molecules with hydrogen bonding. Fig. 3 confirm this fact, as all the SPP-ST_*x*_ nanocomposite membranes showed high proton conductivity at 95% RH compared with 85% RH. The ST perovskite nanoparticles increase the water uptake of the membranes by the formation of proton bonds due to their hygroscopic structure. In fact, nanoparticles form a new direction for proton transport by the retention of water molecules in the membrane. Also, interactions between nanoparticles and polymer create new pathways for protons mobility. Furthermore, the wide surface area of nanoparticles provides active areas for forming more hydrogen bonds.

**Fig. 4:**
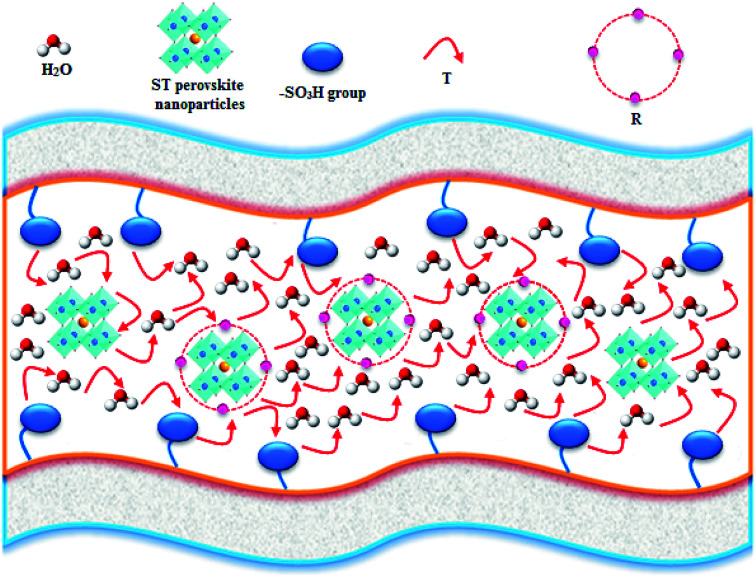
Schemes of proposed proton transport mechanism for SPP-ST_*x*_ nanocomposite membranes.

The ST perovskite nanoparticles have interaction with SPPEK matrix in SPP-ST_*x*_ nanocomposite membranes. Fig. 4 display schemes of proposed proton transport mechanism for SPP-ST_*x*_ nanocomposite membranes.

As can be seen in Fig. 4, proton transfer can happen between adjacent oxygen of the ST perovskite nanoparticles and the –SO_3_H group (active sites) of SPPEK matrix. Proton transfer mechanism in the ST perovskite nanoparticles includes rotational diffusion (R, proton around oxygen) and intraoctahedra proton transfer (T, proton between two adjacent oxygen) in the trap regions.^57,58^ The rotational motion of the proton around oxygen groups is fast.^59^ Hence the reorientation of the proton can happens towards the next oxygen before the transfer.

**Fig. 5:**
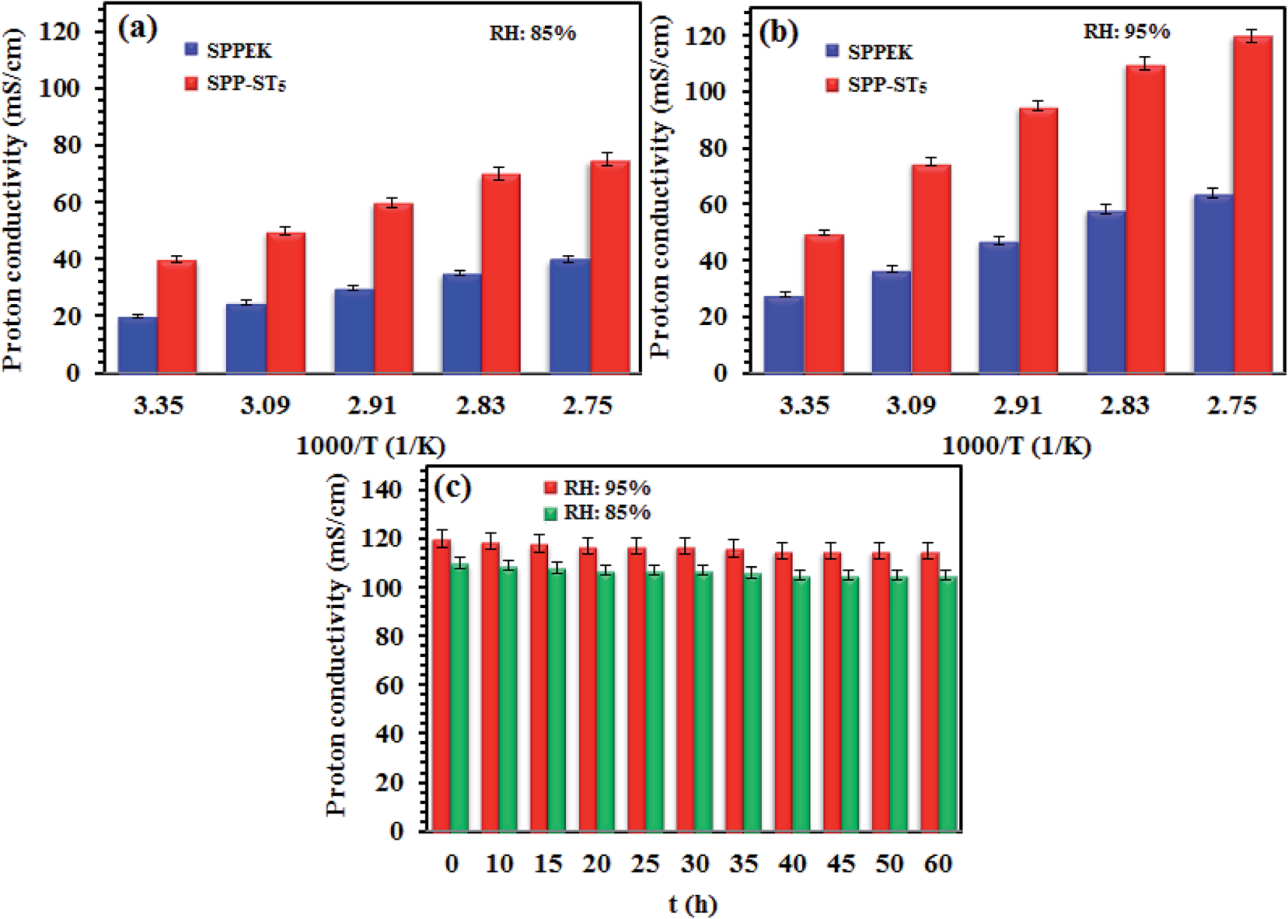
Proton conductivity plots of SPPEK and SPP-ST_5_ membranes in different temperature at (a) 85% and (b) 95% RH. (c) Proton conductivity life time plot of SPP-ST_5_ nanocomposite membranes at 85 and 95% RH and 90 °C.

The proton conductivity of SPP-ST_5_ and pristine SPPEK membranes at different RH and temperatures are shown in Fig. 5. The SPP-ST_5_ nanocomposite membranes showed increasing proton conductivity at higher temperatures due to more mobility of protons. The activation energy (*E*_a_) of proton transport phenomenon can be exported from Arrhenius equation, the slope of the plot ln(*σ*) *versus* 1000/*T* (K). The SPPEK and SPP-ST_5_ membrane exhibits an proton transport *E*_a_ of 7.31 and 5.04 kJ mol^−1^ respectively. The decline of *E*_a_ demonstrated the decreased energy barrier for proton transfer which is due to the formed acid–base interaction between ST perovskite nanoparticles and SO_3_H groups of polymer (faster migration of a high number of protons). So, SPP-ST_5_ nanocomposite membrane has highest potential for use in higher temperature PEM fuel cells due to lowest *E*_a_ value.

Furthermore, flexibility of the hygroscopic domains in SPP-ST_5_ nanocomposite membranes at a higher temperature causes a higher diffusivity of protons. The existence of additional hydrogen bonding network of the ST perovskite nanoparticles is responsible not only for the proton conductivity promotion, but also for the lower activation energy values in the SPP-ST_5_ nanocomposite membranes. The SPP-ST_5_ nanocomposite membranes exhibited high proton conductivity (120 mS cm^−1^) than Nafion 117 (102 mS cm^−1^) at 90 °C and 95% RH. It could be concluded that the water molecules evaporate from Nafion structure quickly at high temperatures and proton conductivity decreases. So SPP-ST_5_ would be a suitable substitution for Nafion, especially at higher temperatures. As can be seen in Table 1, SPP-ST_5_ nanocomposite membranes show high proton conductivity compared with other previous work.

**Table 1:** Proton conductivity of membranes based on SPPEK

Membrane	Temperature (°C)	Proton conductivity (mS cm^−1^)	Ref.
SPP-ST_5_	90	120	This work
M_SSW5_^a^	Ambient	71	50
M_AIT-T_^b^	25	24	52
SPPEK-SGNF^c^	80	104	53
7d^d^	25	1	25
6e^c^^,^^e^	25	10	26
MP10^f^	80	17	27

a Sulfonated poly(vinylidene fluoride-*co*-hexaflouropropylene).

b Fe_2_TiO_5_ modified by 3-aminopropyltriethoxysilane.

c SPPEK doped with sulfonated graphite nanofibers.

d SPPEK with sulfonation degree of 1.2.

e Crosslinked SPPEK.

f SPPEK containing 10% 12-phosphotungstic acid.

Another important factor that affects the efficiency of a membrane as PEM fuel cells is time-stability of proton conductivity. The proton conductivity of the SPP-ST_5_ nanocomposite membranes was investigated by keeping at 80 °C overnight, the results of which are presented in Fig. 5(c). Fig. 5(c) shows the proton conductivity of the SPP-ST_5_ nanocomposite membranes approximately remain constant after 50 h. It indicates the effect of the ST perovskite nanoparticles in the RH preservation and stability of proton conductivity. The oxygen vacancies of the ST perovskite nanoparticles make hydrogen interaction in the membrane and so the adsorbed water amount in the membrane and so the proton conductivity maintains constant.

#### 3.2.3. Water uptake, swelling ratio and ion exchange capacity (IEC)

**Fig. 6:**
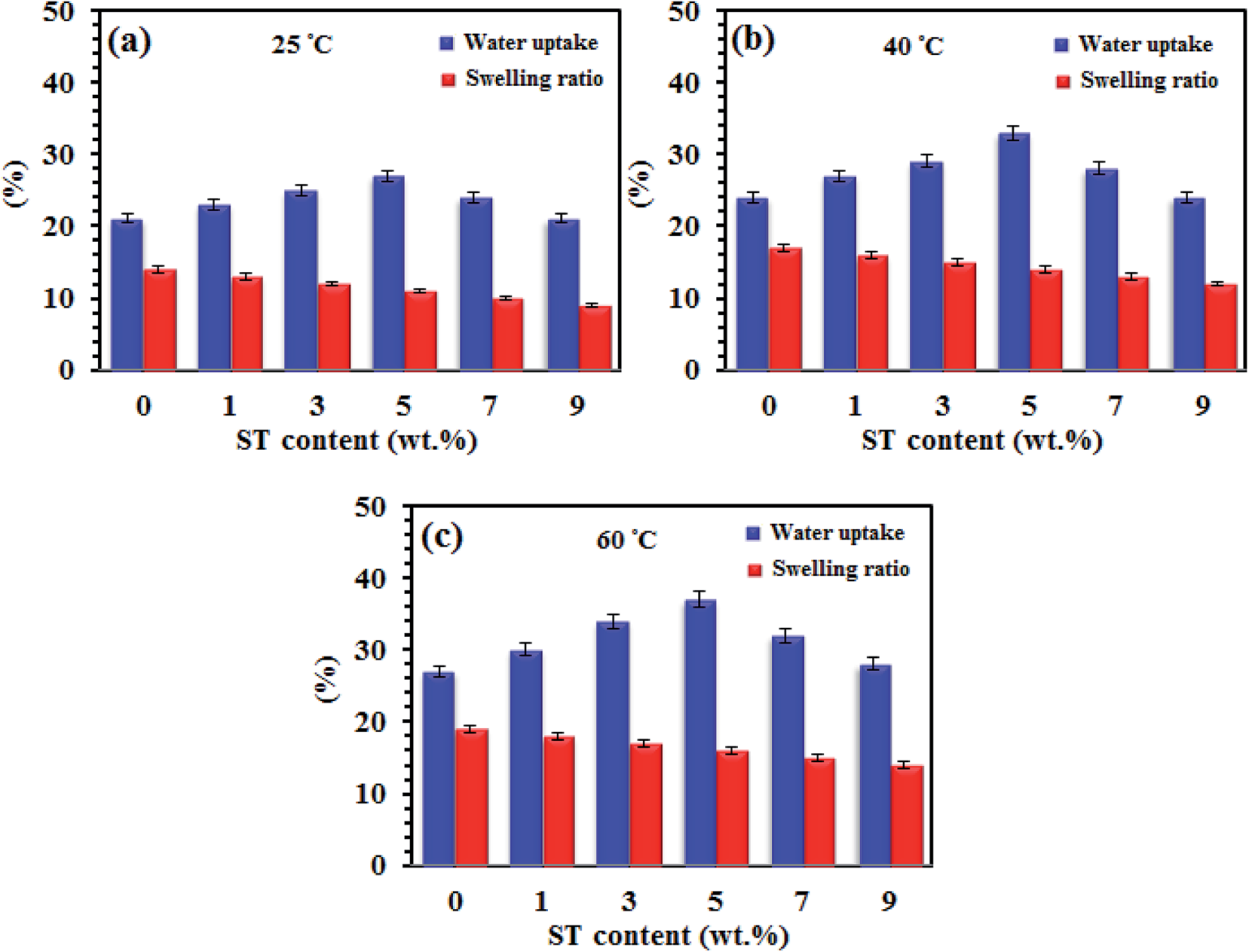
Water uptake and swelling plots (in-plane direction) of SPP-ST_*x*_ nanocomposite membranes at (a) 25 °C, (b) 40 °C and (c) 60 °C.

Fig. 6 demonstrates water uptake and swelling plots of SPP-ST_*x*_ nanocomposite membranes at 25 °C, 40 °C and 60 °C. It is revealed that ST perovskite nanoparticles presence effectively increased the water content of the SPP-ST_*x*_ nanocomposite membranes, which is due to enhancing of the hydrophilicity of the SPPEK matrix.

The oxygen vacancies in the ST perovskite nanoparticles create more sites to absorb free water, formation of hydrogen bonding and so increase the proton conductivity of the SPP-ST_*x*_ nanocomposite membranes. The SPP-ST_5_ nanocomposite membranes displayed higher water uptake (37%) compared with other nanocomposite membranes at 60 °C (Table 2).

**Table 2:** Specification of SPP-ST_5_ and SPPEK membranes

Membrane	Proton conductivity (mS cm^−1^) at 90 °C and 95% RH	Water uptake (%) at 60 °C	Swelling ratio (%) at 60 °C	IEC (meq g^−1^)
SPPEK	50	26	19	1.4
SPP-ST_5_	120	37	14	1.8

Acceptable dimensional stability is a vital parameter of the prepared membranes when used in MEA of PEM fuel cells, which is essential for proper mass transfer and prevention of electrical contact in the MEA. The swelling is an indication to prognosticate the mechanical stability of membranes in PEM fuel cells. Inorganic rigid nanoparticle causes the improvement of mechanical stability of polymeric structures. As shown in Fig. 6, unlike to water uptake, the swelling of the SPP-ST_*x*_ nanocomposite membranes are lower than pristine SPPEK and decrease from 14% to 9% at room temperature with increasing the ST perovskite nanoparticles content, likely due to the compact structure of nanocomposite membranes with formation of hydrogen bonding between ST perovskite nanoparticles and SO_3_H groups of SPPEK membrane. Also, complex structure of SO_3_H– ST perovskite nanoparticles could limit motion of SPPEK chains and has effective effect to constrain the membrane swelling. Thus, nanoparticles make the membranes to be stable mechanically by forming intermolecular interactions and limiting the membranes swelling.^32,37,39^ The results of Fig. 6 shown that both of water uptake and membrane swelling increased with increase temperature. It is maybe due to increase in mobility of free water molecules and SPPEK chains with increasing temperature from 20 °C to 60 °C which facilitates water absorption in polymeric structure.

**Fig. 7:**
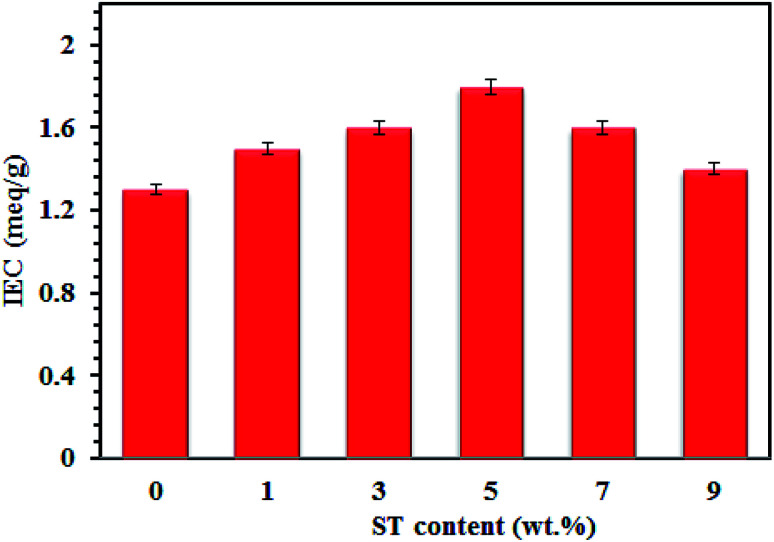
IEC plot of SPP-ST_*x*_ nanocomposite membranes.

Fig. 7 shows that the IEC plot of SPP-ST_*x*_ nanocomposite membranes. The existence of ST perovskite nanoparticles in SPP-ST_*x*_ nanocomposite membranes increases considerably the proton conductivity compared to pure SPPEK membranes.

The IEC values of the nanocomposite membranes increase with increasing the proton conductivity. Increasing the IEC means that there are many sites to transfer the proton. Therefore, the proton conductivity also increases for the nanocomposite membranes with increasing of the IEC value. The SPP-ST_5_ nanocomposite membranes displayed higher IEC (1.8 meq g^−1^) compared to the pure SPPEK and other SPP-ST_*x*_ nanocomposite membranes.

**Fig. 8:**
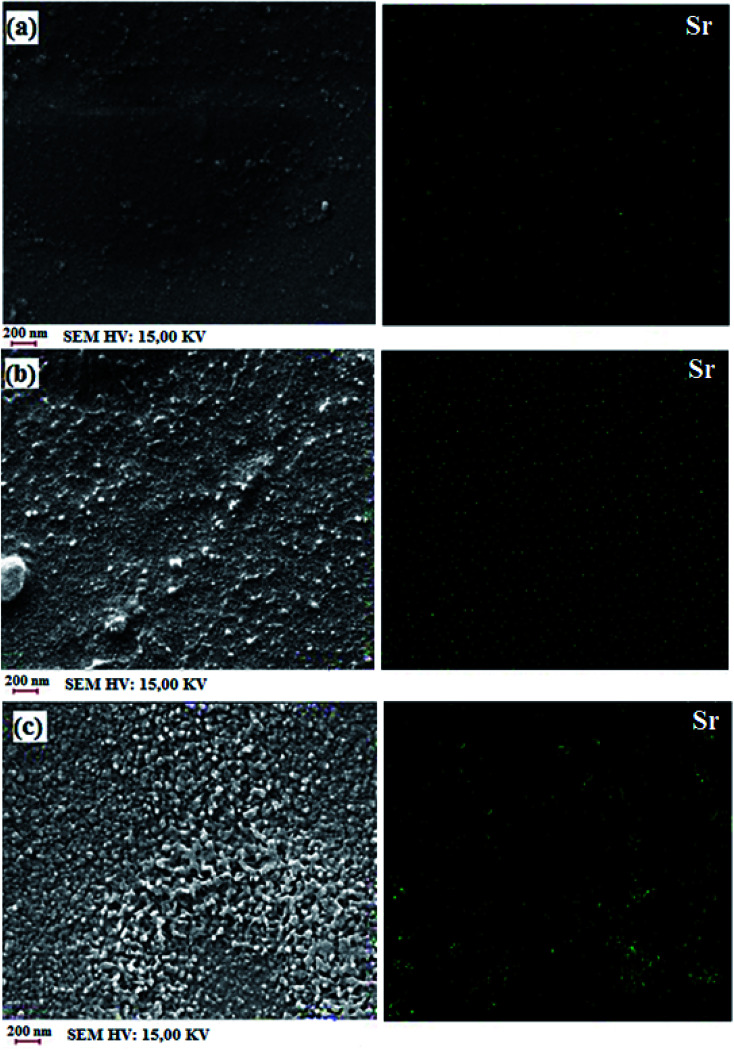
Cross-section SEM-EDX images of SPP-ST_3_ (a), SPP-ST_5_ (b) and SPP-ST_7_ (c) nanocomposite membranes.

Fig. 8 shows the cross-section SEM-EDX images of SPP-ST_3_, SPP-ST_5_ and SPP-ST_7_ nanocomposite membranes. The nanoparticles have high surface energy and so have chemical reactions with the surrounding environment and are not stable. So the high content of the nanoparticles reduces the active surface area and decreases the water uptake and proton conductivity. Another reason for proton conductivity decreases because of the nanoparticle aggregation in the nanocomposite membranes is closing the proton transfer pathways. The high content of nanoparticles would occupy the vacant sites accessible for water molecules in the nanocomposite membranes and decrease the water uptake. So, the amount of the ST perovskite nanoparticles at the preparation process of nanocomposite membranes is an important parameter for controlling the proton conductivity. The ST perovskite nanoparticles (Sr particles) have a homogeneous dispersion in cross-section of the SPP-ST_5_ nanocomposite membranes and the morphology is favorable (Fig. 8(b)). But the morphology of the cross-section of the SPP-ST_7_ nanocomposite membranes due to the agglomeration of the ST perovskite nanoparticles is unfavorable (Fig. 8(c)). The homogeneous distribution of the nanoparticles creates effective hydrogen bonding interactions between the nanoparticles and SPPEK membrane matrix. So, the SPP-ST_5_ nanocomposite membranes have higher proton conductivity and water uptake compared with the SPP-ST_7_ nanocomposite membrane.

#### 3.2.4. Thermal, chemical and mechanical stability

**Fig. 9:**
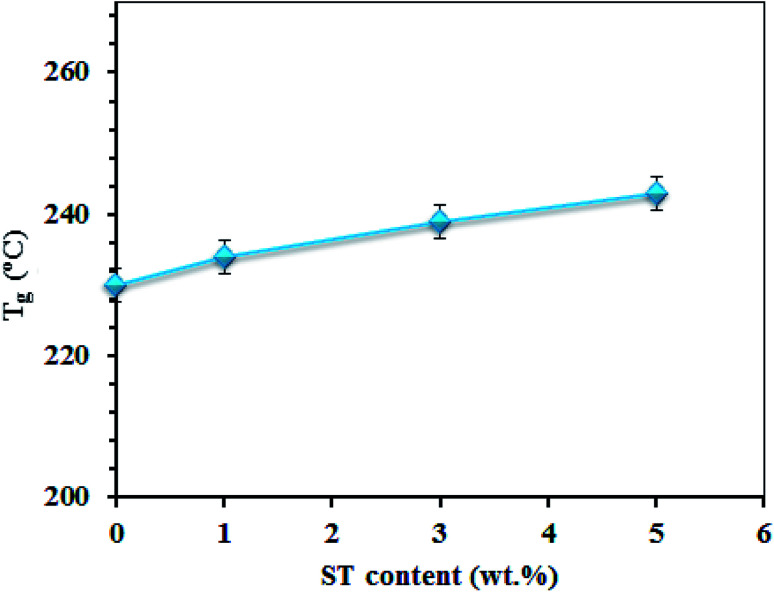
Glass transition (*T*_g_) of SPP-ST_*x*_ nanocomposite membranes.

There are three major steps in TGA plots of SPPEK based membranes. The first step in the TGA plots is a little loss of weight at about 150 °C, which refers to physisorbed water molecules. The second step at about 300 °C indicates the breakdown of SO_3_H groups of SPPEK. And the last step which is a big loss at about 500 °C shows polymer backbone degradation. Fig. 9 displays glass transition (*T*_g_) of SPP-ST_*x*_ nanocomposite membranes. The presence of ST perovskite nanoparticles leads to improvement of the thermal properties of the SPP-ST_5_ nanocomposite membrane. This improvement is due to two reasons; the rigid structure of nanoparticles and the interactions between these nanoparticles and SPPEK backbone.

**Fig. 10:**
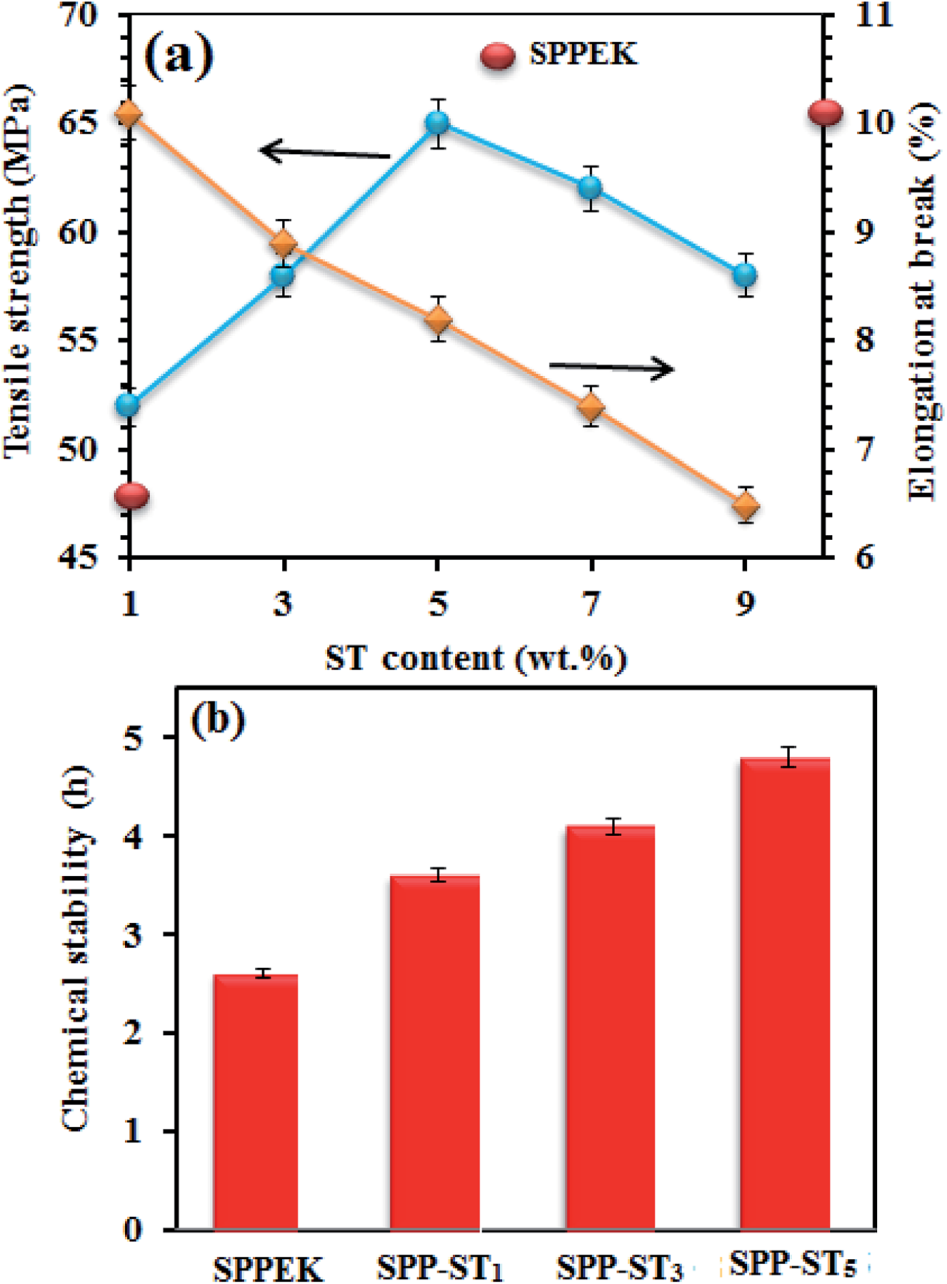
(a) Mechanical stability plot of SPP-ST_*x*_ nanocomposite membranes. (b) Chemical stability plot of SPPEK and SPP-ST_5_ membranes.

Mechanical properties of the SPP-ST_*x*_ nanocomposite membranes are shown in Fig. 10(a). The nanocomposite membrane showed high mechanical stability than pristine SPPEK membrane. The mechanical stability of nanocomposite membranes had picked up due to the strong hydrogen bonds between ST perovskite nanoparticles and SO_3_H groups in SPPEK matrix. The EDX image of SPP-ST_5_ nanocomposite membranes showed that the ST perovskite nanoparticles dispersed in SPPEK matrix homogenously. Therefore, the strong interaction between the nanoparticles and SPPEK was supported resulted in better mechanical stability for SPP-ST_5_ nanocomposite membranes. According to EDX results, agglomeration of nanoparticles in higher content of ST perovskite nanoparticles (>5 wt%) caused weaker mechanical stability. As expected, because of rigid and inflexible nature of ST perovskite nanoparticles, SPP-ST_*x*_ nanocomposite membranes showed lower elongation at break compared with SPPEK.

The effect of the presence of ST perovskite nanoparticles on oxidative stability of membranes, an indication of the membranes' lifetime in the fuel cell was measured by soaking in Fenton reagent. The chemical stability of the membranes was calculated by determining the weight loss in Fenton's reagent. The chemical stability data during 5 h are presented in Fig. 10(b) shows the oxidation stability plot of SPPEK and SPP-ST_5_ membranes. It was expected that the oxidative resistance decrease by increase of water uptake, because the peroxide groups would react with polymer easier in the presence of water. But the reverse results were obtained for SPP-ST_5_ nanocomposite membranes.

The oxidative resistance of SPP-ST_5_ nanocomposite membranes had improved compared with SPPEK membrane, although the water uptake had risen. In other words, the ST perovskite nanoparticles cause better oxidative stability and consequently, longer lifetime of membranes. The nanoparticles cause better oxidative stability and consequently, longer lifetime of membranes by protecting the aromatic structure of SPPEK from meeting the hydrogen peroxide. Strong hydrogen bonds between ST perovskite nanoparticles and SPPEK structure caused the membrane to be more condensed and conserve amine part of the polymer from oxidative attack. In addition, the presence of ST perovskite nanoparticles caused more quantity of SPPEK chains to be refuge.

#### 3.2.5. Fuel cell performance

**Fig. 11:**
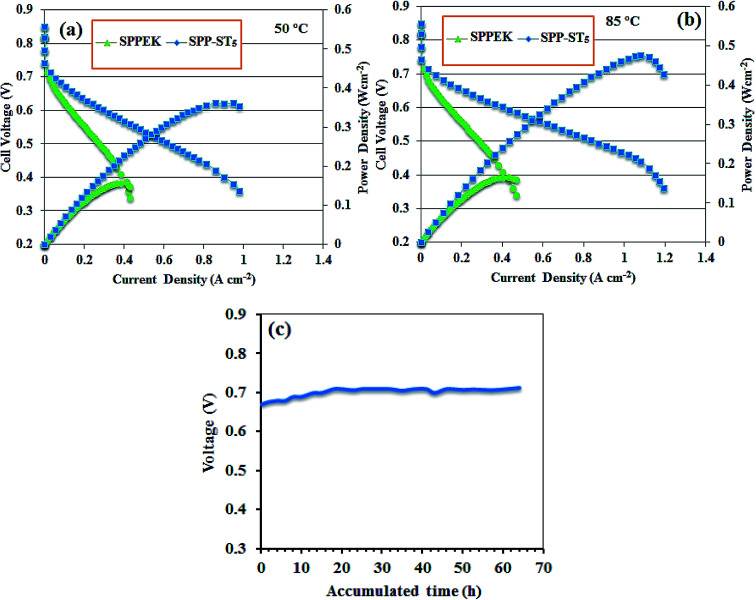
Polarization curves of the SPPEK and SPP-ST_5_ membranes at 50 °C (a) and 85 °C (b) and 95% RH with the flow rates of 120 and 300 mL min^−1^ for H_2_ and O_2_, respectively. (c) Fuel cell life time plot of SPP-ST_5_ nanocomposite membranes at 80 °C and 95% RH.

The SPP-ST_5_ and pristine SPPEK membrane was chosen for fuel cell performance investigation. The prepared MEAs were at a constant potential of 0.5 V for 3 h to chive high fuel cell performance. The flow rates of H_2_ and O_2_ were constant at 120 and 300 mL min^−1^, respectively. Fig. 11 displays polarization curves of SPP-ST_5_ and pristine SPPEK membrane at 50 and 85 °C and 95% RH. The SPP-ST_5_ nanocomposite membranes demonstrated high fuel cell performance compared with pristine SPPEK membrane. The fuel cell performance enhanced with increasing temperature due to the higher proton conductivity in higher temperature which can completely affect the slope of the *I*–*V* curve. The current density of SPP-ST_5_ nanocomposite membranes (at 0.5 V) at 50 °C (Fig. 11(a)) and 85 °C (Fig. 11(b)) were obtained 0.61 mA cm^−2^ and 0.83 mA cm^−2^ respectively. The results suggest that the SPP-ST_5_ nanocomposite membranes have strong potential for use as PEM in PEM fuel cells.

Fig. 11(c) shows the lifetime plots of the SPP-ST_5_ nanocomposite membranes at 80 °C and 95% RH. Fig. 11(c) shows that the lifetime of the SPP-ST_5_ nanocomposite membranes maintain constant for 60 h. The results exhibited that the SPP-ST_5_ nanocomposite membranes proved a suitable fuel cell performance. This is an approval for using the ST perovskites nanoparticles in the PEM for fuel cell applications.

## 4. Conclusions

The potential of SPPEK based nanocomposite membrane in PEM fuel cell applications has been considered in this work with the incorporation of ST perovskite nanoparticles as filler. The nanocomposite membranes showed high water uptake of 37% (at 60 °C) and proton conductivity of 120 mS cm^−1^ (at 90 °C and 95% RH) compared pure SPPEK due to hygroscopic nature of SrTiO_3_ perovskite nanoparticles. The MEA consisting of nanocomposite membrane with 5 wt% ST perovskite nanoparticles gained the power density of 0.41 W cm^−2^ at 85 °C. The results show that nanocomposite membranes are suitable candidate for application in PEM fuel cells.

## Conflicts of interest

There are no conflicts to declare.

## Supplementary Material
